# The Cause–Effect Dilemma of Hematologic Changes in COVID-19: One Year after the Start of the Pandemic

**DOI:** 10.3390/hematolrep14020014

**Published:** 2022-03-28

**Authors:** Ilham Youssry, Dalia Abd Elaziz, Nardeen Ayad, Iman Eyada

**Affiliations:** 1 Pediatric Hematology and BMT Unit, Cairo University, Giza 12613, Egypt; ilhamyoussry@kasralainy.edu.eg; 2 Pediatric Department, Faculty of Medicine, Cairo University, Giza 12211, Egypt; dr_dalia2010@live.com (D.A.E.); imaneyada64@gmail.com (I.E.)

**Keywords:** COVID-19, hematological changes, thrombosis, hematological diseases

## Abstract

COVID-19 is a systemic infection that leads to multisystem affection, including hematological changes. On the other hand, the patients who have certain hematological diseases are more susceptible to COVID-19 infection. The aim of this review is to examine the wide spectrum of hematological changes that are reported to occur due to COVID-19 infection. Most of the studies over the past year mainly show that most of these changes are mainly non-specific, but are of prognostic value. On the other hand, the susceptibility of hematological patients to COVID-19 infection and complications remains questionable. Patients with certain hematological diseases (including malignancy) and those who are treated by aggressive immunosuppressive therapy have shown higher rates of COVID-19 infection and complications. On the other hand, for most of the patients suffering from other chronic hematological conditions, no evidence has shown a greater risk of infection, compared to the general population.

## 1. Introduction

COVID-19 is a systemic infection that leads to multisystem affection, including hematological changes. On the other hand, patients who have certain hematological diseases are more susceptible to COVID-19 infection [1].

The emergence of severe acute respiratory syndrome coronavirus 2 (SARS-CoV-2), the disease named coronavirus disease-19 (COVID-19) [2], resulted in >75 million infections and >1.7 million deaths worldwide, until December 2020 [3]. Coronaviruses are a heterogeneous group of single-stranded plus-sense RNA viruses belonging to the Coronaviridae family and Nidovirales order [4]. The clinical phenotypes of COVID-19 are variable, ranging from asymptomatic up to severe illness with mortality. There are different theories about the variability of the clinical severity among the infected individuals. Those theories can be explained by genetic and epigenetic processes that include high viral load exposure; environmental conditions, such as the absence or presence of an element in the air inhaled by the patient; climate change; pollution, which may aggravate the illness; and the somatic transformation of cells, which occurs in infected human hosts. Finally, severe COVID-19 in previously healthy individuals may result from monogenic predisposition, monogenic inborn errors of immunity of Toll-like receptor 3 (TLR3), and type I IFN cell-intrinsic immunity [5,6].

COVID-19 is a systemic infection that has variable symptoms, which include many respiratory symptoms that can develop into acute respiratory distress syndrome, metabolic acidosis, septic shock, and coagulation dysfunction. Multiple organ failure, including disseminated intravascular coagulation (DIC), can further develop into the occurrence of macrophage activation syndrome (MAS), also known as secondary hemophagocytic lymphohistiocytosis (sHLH) [7,8].

The hematological presentations in COVID-19 are variable and non-specific. While in adults, lymphopenia, leukocytosis or leucopenia, and thrombocytopenia are all reported. in children, the observed hematological abnormalities are non-significant, and the full blood cell counts are normal in most of the patients [9]. 

In this review, we present the interrelation between hematology and COVID-19 infection in its two sides. From one side, we present the hematological presentations and its relation to the disease severity and outcome, and from the other side, we present the susceptibility of patients with hematological diseases to COVID-19 infection.

## 2. COVID-19 Infection of Patients with Hematological Diseases

### 2.1. Cancer Patients and COVID-19 Infection

The highest incidence of complications among patients with hematological diseases in the era of COVID-19 are those with malignancies [1,10]. These complications include a severity of COVID-19 disease, ICU admission, the need for mechanical ventilation, and mortality [10]. The cascade of disease progression among this vulnerable subpopulation is more aggressive and life threatening. Unique pathophysiological changes are still under examination, but several studies have shown that prognostic biomarkers, such as CRP, D-dimer, prothrombin time, and serum IL-6 levels, were significantly higher in cancer patients than in non-cancer patients [11]. More specifically, the concurrence of acute myeloid leukemia in children with COVID-19 presents an extraordinary challenge, since this specific group of patients suffer from a profound and long-lasting humoral and cellular immune deficiency [11,12]. This is illustrated by the fact that the cancer institutes’ guidelines in some countries have recommended delaying the therapy for AML until symptoms resolve and PCR becomes negative [11]. Other studies show that hematopoietic stem cell transplantation (HSCT) (for hematopoietic malignancy and aplasia) or CAR-T therapy have faced unprecedented challenges for fear of infection of the donor, recipient, and healthcare workers [12]. In addition, the elevated number of leukocytes observed in patients with proliferative chronic myelomonocytic leukemia (CMML) increases the risk of leukemoid reactions and overlap syndromes (MDS/MPN). Thus, when they are subjected to COVID-19 infection, they would need a stronger form of immunosuppression, such as hydroxycarbamide, even if they are asymptomatic [13]. On the other hand, results from smaller cancer centers [14] reported successes following a non-myeloablative conditioning regimen followed by post-transplant cyclophosphamide (PTCy), because, in such cases, the successful prophylaxis against graft-versus-host disease (GVHD) could simultaneously work on the attenuation of COVID-19 disease. Moreover, another single-center study demonstrated provisional data regarding the mortality of organ transplant patients that is close to the previous rates before the pandemic, and they suggested that “such patients with an already immune suppressed immune system are not able to produce a cytokine storm and thus do not experience fulminant COVID-19 infection” [15]. What adds to the complexity of the problem, as mentioned by Papakonstantinou et al. [16], is that the molecular mechanisms governing the pathogenesis, metastasis, and relapse of cancer vary widely across the hematological malignancies. Other challenges include the deficiency of cancer research during the COVID-19 pandemic, namely, the strict public health measures taken by authorities to limit the spread of the virus negatively influenced cancer research centers due to quarantining measures, the lack of supplies, and interrupting researchers called to perform emergency hospital duties [17].

### 2.2. Hemoglobinopathies and COVID-19 Infection

In hemoglobinopathies patients, such as sickle cell disease and thalassemia, infection is considered as one of the leading causes of mortality [18,19]. The reason for the higher susceptibility is the comorbidities, including ineffective erythropoiesis, chronic hemolytic anemia, iron overload, and hypercoagulability, which make them vulnerable to complications of COVID-19 infection [19]. Although splenectomy (or auto-splenectomy in the case of SCD) is not known to increase the risk of severe viral infections, splenectomized patients may be at risk of severe secondary bacterial infections when infected by COVID-19 [18]. Although the experience from the 2009 H1N1 influenza pandemic has presented the H1N1 influenza virus as a trigger for acute chest syndrome (ACS) and the need for intensive-care support [18,20], most of the COVID-19 chronic hemoglobinopathy patients have a milder form. Only in a minor percentage of patients can the more severe forms of COVID-19 pneumonia be attributed to the states of hypoxia and VQ mismatch, which sometimes complicate the normal disease course [18,21]. Another consideration is the concurrent mismatch between blood demand and supply in healthcare services during the pandemic, which led to the prioritization of the needs of chronic hemolytic anemia patients [21].

### 2.3. Other Hematological Diseases and COVID-19 Susceptibility

Remarks about other specific hematological diseases in the era of COVID-19 have been made in different settings, but further research is needed to confirm the findings [22,23]. These observations have shown that the prevalence of COVID-19 among congenital bleeding disorders (such as Hemophilia A, von Willebrand disease (vWD)) seems to be low, when compared to the general population [24]. The reason is not yet fully known, but it might be due to the constant vigilance of patients implemented by hemophilia centers, or may be due to the spread of the home replacement therapy [21,23]. Moreover, a severe hypocoagulability state may be protective against COVID-19 hypercoagulability-related adverse effects in the absence of other co-morbidities [23]. Similarly, patients with ITP do not show increased rates of infection, even with low doses of immunosuppressants [23,25]. However, higher doses of steroids and rituximab may need to be avoided during the pandemic, and patients may benefit by attempting to replace them with thrombopoietin receptor agonist (TPO) agents and/or IVIG [25]. Moreover, a study by Quinti et al. [26] reported that certain patients with agammaglobulinemia infected by the virus had a mild clinical presentation of the disease, and, moreover, they did not require immune-modulating drug-blocking IL-6. This was explained by the hypothesis that the lack of B-cell-derived IL-6 levels resulted in an attenuation in the level of inflammation and cytokine storm, which, in return, caused a more favorable outcome than was expected. A similar observation was noted by Soresina et al. [20], who reported two COVID-19 patients with X-linked agammaglobulinemia (XLA); both recovered without the need for intensive care, which is considered as rather atypical for immunodeficiency patients. On the contrary, this cytokine inflammatory storm might not act in the favor of other hematological diseases prone to thrombosis. There are some reports that show that COVID-19-positive patients have higher levels of antiphospholipid antibodies in their blood [23,25]. When trying to quantify the types of antiphospholipid antibodies that mostly increase in this disease, Harzallah et al., from France, reported that, out of 56 patients with COVID-19, 25 cases (45%) were lupus anticoagulant (LAC) positive, in comparison to only 5 out of 50 anticardiolipin antibodies (aCL) or aβ2GPI. The scarcity of clinical follow-up information regarding the thromboembolic phenomenon in these patients [27] mandates the need for further research in this area.

## 3. Hematological Presentations of COVID-19 Infection

### 3.1. Hypercoagulability and the D-Dimer

Arterial and venous thrombosis were correlated with COVID-19 infection. The elevation in D-dimer levels [28], prolongation of prothrombin time (PT), and thrombocytopenia [29] are considered as important markers to assess the severity and outcome of COVID-19 infection. However, these markers may remain normal in many patients, suggesting an unusual prothrombotic state that is distinct from the sepsis-induced coagulopathy [30]. Cytokine storm, hypoxic vaso-occlusion, and the direct activation of immune and vascular cells by viral infection can be potential causes of the macro/micro vascular complications. Additionally, cellular remnants from the neutrophil extracellular traps (NETs) witnessed in many hospitalized patients [31,32] may contribute to the prothrombotic cascade [33]. Furthermore, there are findings that suggest that half of the patients hospitalized with COVID-19 become at least transiently positive for aPL antibodies, including anticardiolipin, anti-β2 glycoprotein I, and anti-phosphatidylserine/prothrombin (aPS/PT), which are potentially pathogenic [34]. The dysregulation of the renin–angiotensin–aldosterone system (RAAS), which induces acute lung injury, further induces endothelial dysfunction causing widespread immune thrombosis and multi-system organ damage [28].

Hence, several risk assessments models (RAMs) and antithrombotic therapeutic options have been tried, and one of the earliest evidence-based WHO guidelines [35] recommended the use of low molecular weight heparin LMWH, which improved 28-day overall survival (one of the earliest applications in a Chinese study [36]). On the other hand, less evidence is found concerning vaccine-induced immune thrombotic thrombocytopenia (VITT), and the issue of anticoagulation with or without the use of IVIG still remains controversial [7]. Some authors have even argued that the addition of fibrinolytic agents to heparin can increase the incidence of bleeding and, in this case, the improvement in the D-dimer can exceptionally be used as a biomarker of fibrinolysis suppression in high-risk patients [37].

Studies demonstrated other changes in coagulation (apart from D-dimer elevation), such as the prolongation of prothrombin time and activated thromboplastin time, which indicate laboratory coagulopathy that progresses to clinically evident disseminated intravascular coagulopathy (DIC) (defined by the International Society on Thrombosis and Haemostasis Score). The progression to DIC in infected patient predicted a poor prognosis, occurring in 71.4% of all non-survivors vs. 0.6% of survivors [28]. 

### 3.2. Complete Blood Counts and Other Laboratory Markers

During the incubation period and the early phase of the disease, peripheral blood leukocyte and lymphocyte counts were normal or slightly reduced. Following viremia, COVID-19 primarily affects the tissues expressing high levels of ACE2; with a pronounced systemic increase in inflammatory mediators and cytokines, significant lymphopenia becomes evident [38]. Thrombocytopenia and leucopenia were also described in several studies, highlighting the association between lymphopenia, the need of ICU admission, and the development of acute respiratory distress syndrome (ARDS) [38,39].

Several theories explained the cause of this lymphopenia and included the presence of the ACE2 receptor on the lymphocytes’ surface, leading to their lysis [40]; Bo Diao et.al reported that the decrease in the numbers of T-lymphocyte (CD3^+^, CD4^+^, and CD8^+^) subsets was inversely proportionate to the inflammatory cytokines, including IL-6, IL-10, and TNF-a, which promote lymphocyte apoptosis [41]. Furthermore, the coexistence of lactic acid acidosis may also be an adding factor that inhibits lymphocyte proliferation [42] Thrombocytopenia is also significantly associated with the intensity of the COVID-19 disease [38]. Leukocytosis with neutrophilia were significantly reported in patients with myocardial injury admitted in ICU, compared to the others [43,44]. In a recent study conducted by Urbano et al. [45] in 2022, interesting results reported a 16-times-higher risk of mortality in patients with a neutrophil count of 5.91 × 109/L or higher, compared to normal levels. Nevertheless, both can be an indication of the coexistence of bacterial infection [38,46]. Additionally, neutrophilia could be an expression of the cytokine storm and hyper-inflammatory state [47,48,49,50]. A serial assessment of the counts level presents a predictive value; the greater the change in the counts over time, the more severe the disease, resulting in deterioration and the necessity of ICU hospitalization [39,48,51,52].

### 3.3. The Biomarkers Procalcitonin, Ferritin, and C-Reactive Protein, IL6

1. C-reactive protein (CRP) is an acute phase reactant. Its high level is associated with admissions to the ICU, ARDS development, and not significantly associated with mortality [43]. On the other hand, Deng Y and his colleagues reported that it was significantly higher in the death group than the recovered group [53]. It could also indicate the associated bacterial infection.
2. Procalcitonin is a precursor of calcitonin, a hormone that plays a pivotal role in calcium homeostasis. In severe COVID-19 infections, which requires ICU admission, high levels of procalcitonin were observed [54], which could be explained by the association of bacterial infection.
3. IL-6: COVID-19 infections can cause cytokine storm and macrophage activation syndrome (MAS), due to the hyper-activated T lymphocytes with the release of different inflammatory cytokines, including IL-6 [55,56]. Based on this pathophysiology, Tocliziumab, which is a recombinant humanized monoclonal anti-IL-6 receptor antibody, inhibiting IL-6, which has been used in auto-inflammatory diseases [56], can be tried. Tocliziumab is the best-studied drug, as along with sarilumab, among IL-6 inhibitors in COVID-19 patients, showing promising results among critically ill patient with MAS [57,58].

Unlike in the case of adults, the radiological and laboratory findings were non-specific in children, and the full blood cell counts were normal in the majority of patients, while the CRP and pro-calcitonin were abnormal in almost one third of the patients [8]. In pediatric inflammatory multisystem syndrome temporally associated with COVID-19 (PIMS-TS) or multisystem inflammatory syndrome in children (MIS-C), there were neutrophilia, abnormal levels of CRP, ESR, ferritin, and D-dimers. The studies supported that high WBCs, neutrophils, CRPs, low lymphocytes, anemia, low platelets, high fibrinogen levels, procalcitonin, ALTs, and Troponin are more commonly associated with MIS-C rather than Kawasaki disease (KD), while the D-dimer level was similar between MIS-C and KD [59,60,61]. Despite the high levels of D-dimers among the hospitalized children, the studies did not point to the use of an anticoagulant in these children with a very good outcome and discharge from the hospitals [59,60,61].

## 4. Conclusions

Patients with certain hematological diseases (including malignancy) and those who are treated by aggressive immunosuppressive therapy have shown higher rates of COVID-19 infection and complications. On the other hand, for most of the patients suffering from other chronic hematological conditions, no evidence has shown a greater risk of infection, compared to the general population. However, COVID-19 leads to a wide spectrum of hematological changes that are non-specific, but are of prognostic value.

## Data Availability

No data reporting.
